# A Novel Flow Cytometric HTS Assay Reveals Functional Modulators of ATP Binding Cassette Transporter ABCB6

**DOI:** 10.1371/journal.pone.0040005

**Published:** 2012-07-10

**Authors:** Kishore Polireddy, Mohiuddin Md. Taimur Khan, Hemantkumar Chavan, Susan Young, Xiaochao Ma, Anna Waller, Matthew Garcia, Dominique Perez, Stephanie Chavez, Jacob J. Strouse, Mark K. Haynes, Cristian G. Bologa, Tudor I. Oprea, George P. Tegos, Larry A. Sklar, Partha Krishnamurthy

**Affiliations:** 1 Department of Pharmacology, Toxicology, and Therapeutics, The University of Kansas Medical Center, Kansas City, Kansas, United States of America; 2 Center for Molecular Discovery, University of New Mexico, Albuquerque, New Mexico, United States of America; 3 Division of Biocomputing, University of New Mexico Health Sciences Center, Albuquerque, New Mexico, United States of America; 4 Department of Pathology, University of New Mexico School of Medicine, Albuquerque, New Mexico, United States of America; 5 Wellman Center for Photomedicine, Massachusetts General Hospital, Boston, Massachusetts, United States of America; 6 Department of Dermatology, Harvard Medical School, Boston, Massachusetts, United States of America; Enzo Life Sciences, Inc., United States of America

## Abstract

ABCB6 is a member of the adenosine triphosphate (ATP)-binding cassette family of transporter proteins that is increasingly recognized as a relevant physiological and therapeutic target. Evaluation of modulators of ABCB6 activity would pave the way toward a more complete understanding of the significance of this transport process in tumor cell growth, proliferation and therapy-related drug resistance. In addition, this effort would improve our understanding of the function of ABCB6 in normal physiology with respect to heme biosynthesis, and cellular adaptation to metabolic demand and stress responses. To search for modulators of ABCB6, we developed a novel cell-based approach that, in combination with flow cytometric high-throughput screening (HTS), can be used to identify functional modulators of ABCB6. Accumulation of protoporphyrin, a fluorescent molecule, in wild-type ABCB6 expressing K562 cells, forms the basis of the HTS assay. Screening the Prestwick Chemical Library employing the HTS assay identified four compounds, benzethonium chloride, verteporfin, tomatine hydrochloride and piperlongumine, that reduced ABCB6 mediated cellular porphyrin levels. Validation of the identified compounds employing the hemin-agarose affinity chromatography and mitochondrial transport assays demonstrated that three out of the four compounds were capable of inhibiting ABCB6 mediated hemin transport into isolated mitochondria. However, only verteporfin and tomatine hydrochloride inhibited ABCB6’s ability to compete with hemin as an ABCB6 substrate. This assay is therefore sensitive, robust, and suitable for automation in a high-throughput environment as demonstrated by our identification of selective functional modulators of ABCB6. Application of this assay to other libraries of synthetic compounds and natural products is expected to identify novel modulators of ABCB6 activity.

## Introduction

Transporters perform essential roles in cellular metabolism and activity. They differ in membrane topology, energy coupling mechanisms, and most importantly in substrate specificities [1]–[4]. Based on their sequence similarity and structural homology, transporters are classified into six super-families [1], [3]. The ATP binding cassette transporter superfamily is the largest, comprising seven subfamilies designated A to G [1], [4]. ABC transporters are increasingly recognized as playing important roles in normal biology and therapeutic responses to medications in mammalian cells. The highly conserved ABC domains of ABC transporters provide the nucleotide-dependent engine that drives transport [1], [2].

ABCB6 belongs to the B sub-family of ABC transporters, which includes the well-characterized human transporter ABCB1 that was the first ABC transporter implicated in multidrug resistance, the intracellular peptide transporters (TAP1 and TAP2) that function in major histocompatibility complex class I antigen presentation and ABCB5 which is essential for melanoma induction, as a doxorubicin efflux mediator in melanomas and xenotransplantation proliferation models [3], [5]. ABCB6 is increasingly recognized as a relevant physiologic and therapeutic target. ABCB6 expression is upregulated in many tumor cell lines and in liver tumors where it appears to promote cell survival and tumor growth and proliferation [5]–[7]. ABCB6 gene is amplified in tumor cells with acquired chemotherapeutic resistance [8]–[13]. ABCB6 expression is also induced under cell stress, where it promotes cell survival [8], [14]–[16]. Thus, ABCB6 expression could promote multiple survival strategies that are usually the hallmark of tumor development and progression. Therefore, development of potent and selective chemical probes that can modulate ABCB6 transporter function may have oncologic as well as pharmacologic applications. Development of such modulators would also improve our understanding of ABCB6 substrate specificity and ABCB6 transporter function with regards to heme biosynthesis, mitochondrial function, and cellular adaptation to metabolic demand and stress.

In this report, we describe the development, optimization, and validation of a novel robust high-throughput fluorescence based flow cytometry assay designed to interrogate modulators of ABCB6 activity. We have previously demonstrated that ABCB6 regulates the synthesis and accumulation of the fluorescent compound protoporphyrin (PPIX) [14], [17]. Increased ABCB6 expression in cells selected for over-expression of wild-type ABCB6 results in increased cell-associated PPIX fluorescence intensity while having no effect on the fluorescence properties of the molecule [14], [17]. This has formed the basis of a flow cytometry assay to develop modulators of ABCB6 activity. Using this assay, we have identified and characterized modulators of ABCB6 activity from the Prestwick Chemical Library (PCL). The identified chemotypes may represent leads for the development of novel chemical probes for ABCB6.

## Materials and Methods

### Cells Lines and Culture Conditions

Human Erythroleukemia (K562) cells were obtained from American Type Culture Collection (ATCC, Manassas, VA). K562 cells were engineered to constitutively express either the wild-type ABCB6 or the mutant ABCB6 (under the control of a CMV promoter) as described [14], [17]. These cells exhibit stable expression of ABCB6 for 60 passages (Fig. S1). Cells were cultured as previously described [14] using modified eagle’s medium (CellGro, VA) supplemented with 10% fetal bovine serum (Hyclone, Logan UT) and 100 units/mL penicillin/streptomycin.

### Chemicals

*The Prestwick Chemical Library* (http://www.prestwickchemical.fr/) contains 1120 small molecules, 90% marketed drugs and 10% bioactive alkaloids or related substances with a high degree of drug-likeliness. The active compounds were selected for chemical and pharmacological diversity and their potential for clinical trial drug repurposing since their bioavailability and safety is well documented. The PCL 2000 ® was designed to reduce the risk of “low quality” hits, reduce the cost of the initial screening, and accelerate lead discovery. The 1120 molecule collection of the library supersedes a previous 880 small molecule collection. Tomatine, benzethonium chloride (BEC), verteporfin, piperlongumine, and succinylacetone were subsequently purchased from Sigma Chemical Co (St. Louis Mo).

### HTS Flow Cytometry and Data Analysis

The HyperCyt® flow cytometry platform [18] interfaces a flow cytometer and an autosampler that sequentially aspirates particle suspensions from individual wells or a microtiter assay plate. Between wells, the pump draws a bubble of air into the sample line generating a tandem series of bubble-separated samples for delivery to the flow cytometer. Sample and bubble volumes are determined by the time the autosampler probe is in a well or above a well taking in air. Accurate measurements have been demonstrated in endpoint assays over a range of fluorescence intensities using input cell concentrations of 1–20 million/mL and source well volumes of 5–100 µL. Immediately after data acquisition by the flow cytometer, specialized software (IDLeQuery) was used to analyze the data file. A flow chart describing the general scheme of the HTS assay is presented in Fig. S2.

### General Assay and HTS Conditions

A 10 µL volume of ABCB6 overexpressing or empty vector K562 cells, at a density of 4–5×10^7^ cells were transferred to 384-well microplates containing 10 µL volume of the test compounds in DMSO and 10% serum PBS. Test compounds were diluted in 5% serum-PBS-DMSO to obtain a final concentration of 6.67 µM. DMSO concentration was maintained at 1%, which is well tolerated by the cells and the assay conditions (Fig. S9). Microplates were incubated overnight at 37°C. HyperCyt® sampling consisted of 1–2 µL taken from a 20 µL well volume at room temperature. Data analysis was performed with HyperView software program to automatically detect the time-resolved data clusters (wells) and determine the mean channel fluorescence (MCF) by FL2 (PE) [19]–[21]. Acquired data were automatically exported to Microsoft excel for calculating the assay quality control Z’ as described [22]. The Z’ factor is a statistical parameter utilized in high throughput screening to evaluate the ability of an assay to identify a hit compound. Z’ signal to noise (S/N), and signal to background (S/B) statistics were calculated using the following equation and as described in Zhang et al 1999. Z’  = 1 - (3*STDpos + STDneg)/(ABS[AVEpos-AVneg]). In this equation STDpos is the standard deviation of the positive controls, STDneg is the standard deviation of the negative controls, AVEpos is the mean of the positive controls and AVEneg is the mean of the negative controls. The Abosrobance (ABS) represents the absolute value of the difference between the different control means.

### Plate Configuration and Screening

384-well plates were configured with 32 control wells and 32 buffer wells. Columns 1 and 24 contained the 1% DMSO/buffer (negative control), columns 2 and 23 with ABCB6-mutant cells in 1% DMSO/buffer. This leaves 320 wells (columns 3 - 22) to which PCL test compounds were added. Sampling was started after incubating the plates overnight at 37°C and 5% CO_2_.

### Confirmatory Dose Response Assay

Test compounds (potential hit compounds) displaying activity after the PCL screening were serially 2-fold diluted in a concentration range from 48.8 nM to 100 µM. Assay conditions were identical with the primary HTS assay employing ABCB6 overexpressing and vector cells. Triplicate curves were fitted by Prism software (GraphPad Software Inc., San Diego, CA) using nonlinear least-squares regression in a sigmoidal dose–response model with variable slope, known as the four-parameter logistic equation. Curve fit statistics were used to determine the half-maximal effective concentration (EC_50_ in µM) and the Hill slope of the fitted curve.

### Cell Viability and Cytotoxicity

The luminescent Cell Viability Assay kit *CellTiter-Glo* from Promega (Madison, WI) was used and the assay was performed following the manufacturer’s instruction as described previously [21]. Briefly, at the time of passage, cell suspensions (99 µL, 10,000 cells/well) were added into opaque-walled 96-well plates (Corning, Corning, NY). After stabilization, test compounds were added to the wells at 1 µL per well resulting in a final concentration range from 380 nM to 100 µM. Vehicle control wells contained 1% DMSO. Following treatment, cells were incubated (37°C and 5% CO_2_) for 72 hours prior to adding CellTiter-Glo reagents and followed by a 10 min incubation at room temperature in the dark. Luminescence intensity (LI) was recorded using a Wallac 1420 plate reader (PerkinElmer, Norwalk, CT). The percentage of normalized viable cells was calculated using the following equation: % viability  = 100× (LI experiment - LI background)/(LI vehicle control - LI background).

### Molecular Modeling/Docking Protein Modeling and Docking

The sequence of the human ABCB6 transporter as extracted from the UniProt database [23], [24] (accession code: Q9NP58) was aligned using the T-Coffee package [25] with both the Val33-Thr626 and Leu684-Ala1271 parts of the sequence of the mouse ABCB1a transporter retrieved from the RCSB Protein Data Bank (PDB entry: 3G5U). The alignment was further manually refined in order to remove the insertions and deletions in the alpha helices (Fig. S3). The 3D model of the dimer human ABCB6 transporter was generated using the protein structure modeling package Modeller [26], and was prepared for docking using the standard protocol implemented in Fred_Receptor, a wizard like graphical utility that prepares an active site for docking with FRED (OpenEye Scientific Software Inc. Santa Fe, NM. www.eyesopen.com). Three dimensional (3D) conformations for the chosen ligands (tomatine, verteporfin, benzethonium, piperlongumine, and the endogenous substrate coproporphyrinogen III) were generated using OMEGA (OpenEye Scientific Software Inc. Santa Fe, NM. www.eyesopen.com) and the ligands were docked to the protein with FRED, using the default docking parameters, and a box big enough to include most part of the transmembrane region. The Chemgauss3 scoring function, which uses Gaussian smoothed potentials to measure the complementarity of ligand poses within the active site, was used to evaluate and compare how well the different ligands fit in the receptor binding site.

### Primary Hit Validation Selectivity/specificity and Functional Relevance

Compounds identified to be non-toxic to the primary cells were screened for selectivity/specificity using four different assays.

#### 1) Hemin agarose affinity chromatography

The assay was performed as described previously [17]. Briefly, mitochondrial lysate (20 µg) or purified ABCB6 protein (300 ng for immunoblots and 3 µg for silver staining and coomassie staining) with a flag tag were incubated at room temperature for 15 min in the presence of 167.5 nM hemin-agarose (Sigma, StLouis, MO). The reaction mixture was centrifuged at 4°C. The resulting pellet was washed thrice with 1 mL lysis buffer, resuspended in 20 µL of 2X SDS sample-loading buffer, and then centrifuged. The supernatant from the final spin was analyzed on a 5–20% gradient gel, transferred to a nylon membrane, and probed with a monoclonal antibody to the flag epitope to identify ABCB6-flag (Sigma, StLouis, MO). A schematic representation of the hemin-agarose affinity chromatography assay is presented in Fig. S4.

#### Purification of ABCB6 protein with a flag tag used in hemin agarose affinity chromatography described above

A detailed description of the purification protocol is presented in a manuscript that is currently in preparation. Briefly, HEK293 cells expressing ABCB6-flag (transduced with ABCB6-flag lentiviral particles) were harvested and lysed in lysis buffer (0.2%Triton X 100 in 1X PBS containing 1 mM phenylmehanesulfonylfluoride (PMSF), Pepstatin, Aprotinin and Leupeptin). Following centrifugation at 1,000 rpm for 10 min at 4°C the supernatant was incubated with 1 mL of anti-flag M2 affinity beads (Sigma, StLouis, MO) overnight at 4°C. The antibody-ABCB6-flag complex formed following overnight incubation was washed twice with lysis buffer and centrifuged to remove the supernatant. The antibody-ABCB6-flag-bead complex (pellet) was resuspended in lysis buffer and transferred to handee spin cups (Peirce, Rockford, IL) followed by additional washing steps using lysis buffer and wash buffer (10 mM Tis, 0.2% Triton X-100). ABCB6-flag protein was finally eluted from the column using elution buffer containing Glycine and 0.2% Triton –X-100. The eluted protein was quantified and purity analyzed by SDS-PAGE following coomassie blue staining and silver staining.

#### 2) Mitochondrial transport assay: Hemin competition

The assay was performed as described previously [17]. Briefly, mitochondrial preparations containing 20 µg of protein were incubated with an ATP regenerating mix along with 23 nM Fe^55^-hemin in either the presence or absence of the potential hit compound. The reaction was stopped at the indicated time and filtered through a cellulose filter. The filter was washed and analyzed by liquid scintillation counting. Fe^55^ hemin uptake was defined as the difference between substrate binding to mitochondria at 4°C and after incubation at 37°C in the presence or absence of ATP.

#### 3) Mitochondrial transport assay: verteporfin and tomatine uptake studies

The assay was performed as described above with four modifications; a) to increase the power of the assay to distinguish between ABCB6 dependent and ABCB6 independent transport we performed transport using mitochondria isolated from cells expressing either a transport competent ABCB6 protein (ABCB6-wildtype; referred to as ABCB6) or a transport incompetent ABCB6 protein (ABCB6-mutant; referred to as ABCB6-MT) protein. The ABCB6-MT carries a single aminoacid change in the walker A domain of ABCB6, which is the nucleotide binding domain in ATP binding cassette transporters that is essential for transport function (1, 17); b) the amount of mitochondria used in these assays were 100 µg (mitochondrial protein) instead of the 20 µg used in the competition studies c) in these assays radioactive hemin was omitted and only verteporfin or tomatine hydrochloride were used as substrates, d) following uptake the reaction was stopped at the indicated time and instead of filtering through a cellulose filter, the mitochondria were pelleted at 17,000× g for 10 min at 4°C. The pelleted mitochondria were washed three times with transport buffer. The final pellet was resuspended in 100 µL of acetonitrile and the amount of verteporfin or tomatine taken up into isolated mitochondria was analyzed using Ultra Performance Liquid Chromatography-time-of-flight mass spectrometry (UPLC-TOFMS) as previously described [27]. UPLC takes full advantage of chromatographic principles to run separations using columns packed with smaller particles for superior resolution and signal-to-noise ratio. Increased analyte concentration and reduced chromatographic dispersion achieved by UPLC promote increased electrospray ionization efficiency giving improvements in response and in confirmation of identity. The concentration of verteporfin and tomatine hydrochloride following uptake into mitochondria was calculated from a standard curve generated using the respective compounds.

#### 4) ATPase activity assay

ATPase activity was measured as described previously [17]. Briefly, vanadate-sensitive ATPase activity was measured in crude mitochondrial fraction isolated from K562-ABCB6-flag, and K562-ABCB6-Mutant-flag (K562-MT-flag) cells by colorimetric assay as described [17]. Preparations of the isolated mitochondria containing 30 µg of protein were incubated with the indicated concentrations of verteporfin or tomatine hydrochloride in methanol; the reaction was started by addition of 3.3 mM MgATP. The vanadate-sensitive ATPase activity stimulated by verteporfin and tomatine hydrochloride was measured as nanomoles of inorganic phosphate released per minute per milligram of protein.

## Results and Discussion

### Assay Development and HTS Flow Cytometry

**Figure 1:**
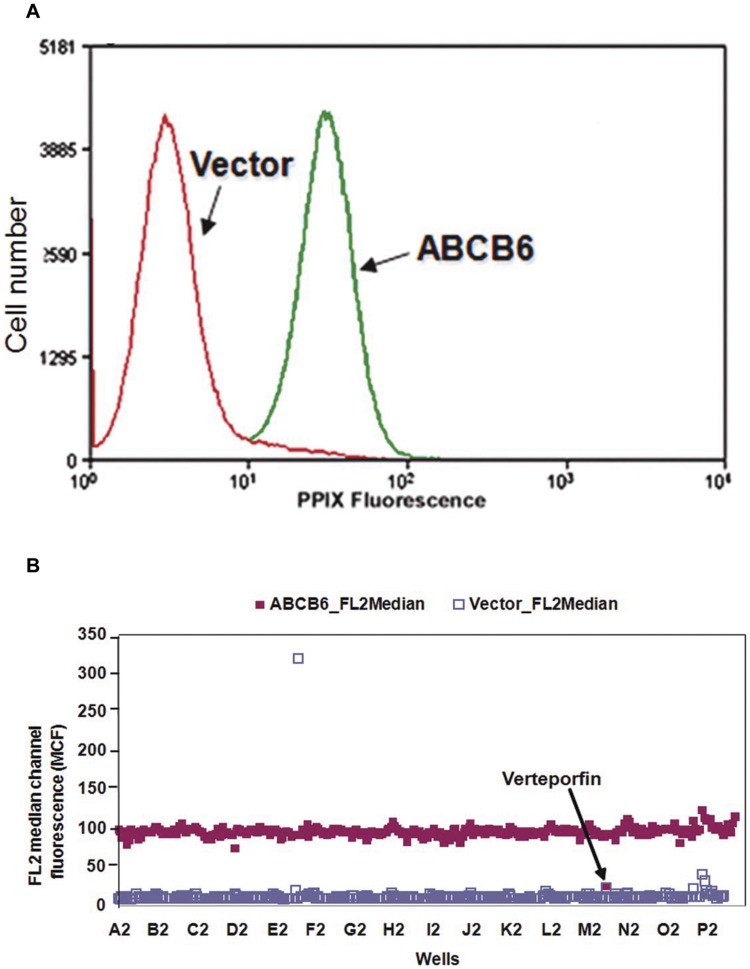
Assay development at University of New Mexico Center for Molecular Discovery (UNMCMD). (a) Human erythroid leukemia cells (K562) expressing ABCB6 accumulate more porphyrins compared to the vector control cells as measured by flow cytometry. (b) Flow cytometric HTS of Prestwick Chemical Library (PCL). The figure depicts data from a representative PCL plate demonstrating decrease in FL2 fluorescence (representing PPIX fluorescence) mediated by verteporfin. The Y-axis displays the amount of fluorescence (FL2 log channel representing PPIX fluorescence). The X-axis displays each well. In the assays time bins were automatically drawn around the clusters by using IDLQuery software programs, each cluster corresponds to one well. The red square represents the PPIX fluorescence in untreated ABCB6 overexpressing cells while the white square represents the PPIX fluorescence in vector control cells. The arrow highlights the decrease in PPIX fluorescence of ABCB6 expressing cells in the presence of verteporfin.

We developed an HTS flow cytometry assay to select functional modulators of ABCB6. The assay takes advantage of the unique ability of ABCB6 to regulate cellular porphyrin biosynthesis [17]. Cells over expressing ABCB6 exhibit increased porphyrin biosynthesis, which directly correlates with the level of ABCB6 expression (Fig. 1a and [17]). Furthermore, the increased cellular porphyrin synthesis requires a functional protein, because a non-functional transport mutant of ABCB6 is incapable of promoting porphyrin biosynthesis [17]. Accumulation of porphyrin in cells results in increased cell associated fluorescence intensity (Fig. 1a). Cellular porphyrin representative of porphyrin levels was confirmed using succinylacetone (SA) as described [14], [17]. SA is a potent inhibitor of cellular porphyrin biosynthesis and we have previously demonstrated that SA at a concentration of 200 µM completely blocks cellular porphyrin biosynthesis in less than 24 hours [14], [17].

Feasibility of the HTS assay to identify compounds that functionally alter ABCB6 mediated porphyrin biosynthesis was evaluated using the PCL 2000 library. Although the central idea was to evaluate compounds that decreased the median channel fluorescent (MCF) intensity by FL2 (Fig. 1b, Fig. S5a, S5b and S5c), the assay also identified compounds that increased the MCF intensity (Fig. S5c) suggesting that the assay is capable of identifying both inhibitors and activators of ABCB6. The controls were K562 cells overexpressing ABCB6 treated with DMSO alone (negative control). Our analysis showed a tight data distribution with Z’ factors averaging 0.61+/−0.08 and a hit rate of 0.4%. Calculations of percentage response were set to 100% at the positive control level and 0% at the negative control level. Cutoffs were set at three times higher than the standard deviation of mean fluorescence values, which corresponded to a percentage response of 58%.

To minimize false positive hits we selected only compounds that satisfied the cutoff criteria because they concurrently decrease fluorescence. By using the criteria used in FL2, 12 compounds from the entire PCL set qualified as hits. However, 8 of these compounds were flagged as potentially fluorescent as the FL2 MCF in the vector was greater than average +2X standard deviation of DMSO control (which corresponded to the DMSO control value measured in the ABCB6 cells). Four compounds met the criteria (0.4% hit rate). The identified compounds were verteporfin, benzethonium chloride, tomatine hydrochloride and piperlongumine (Fig. 1b and Fig. S5a, S5b, S5c and S6).

### Confirmatory Dose Responses

**Figure 2:**
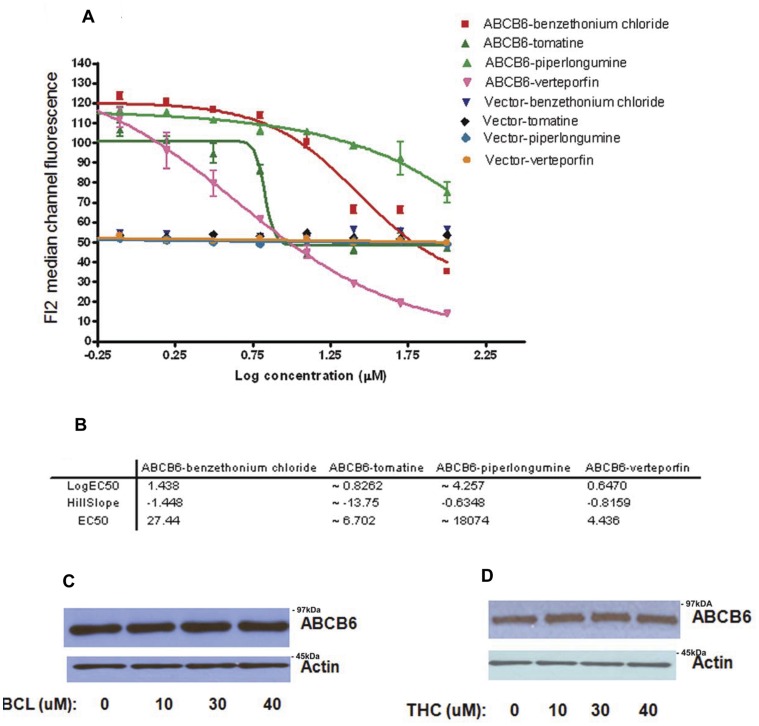
Dose response analysis of active compounds identified in the primary HTS. (a) ABCB6 expressing cells showed dose dependent decrease in median channel fluorescence (representing PPIX fluorescence) in response to benzethonium chloride, verteporfin, and to a lesser extent piperlongumine. In contrast vector control cells did not show any change in median channel fluorescence of FL2 (representing PPIX fluorescence) in response to treatment with any of the four compounds. (b) EC_50_ values for each of the compound and their corresponding hillslope values. Results represent mean +/− SD with n = 3. (c and d) compounds identified in the primary HTS screen do not alter ABCB6 expression in the ABCB6 over-expressing cells. (c) immunoblot analysis of ABCB6 expression in ABCB6 overexpressing cells treated with increasing concentration of benzethonium chloride (BCL). (d) immunoblot analysis of ABCB6 expression in ABCB6-flag overexpressing cells treated with increasing concentration of tomatine hydrochloride (THC). In these studies ABCB6 expression was measured using a monoclonal antibody against the flag-tag. Actin is used a loading control. Figure representative of three independent experiments.

Compounds that displayed activity in the primary HTS assay were re-tested for dose response. The four hit compounds that decreased MCF intensity by FL2 in the primary screen were evaluated employing *A*BCB6 over-expressing and vector cells to identify compounds decreasing MCF by FL2 in a dose response manner (dose concentration ranging from 48.8 nM to 100 µM). All the four compounds (verteporfin, benzethonium chloride, tomatine hydrochloride and piperlongumine) showed a dose dependent ability to reduce ABCB6 mediated PPIX accumulation (Fig. 2). In contrast, none of the compounds had any effect on vector cells. Verteporfin exhibited the lowest EC_50_, while piperlongumine exhibited the highest. Interestingly an increase in tomatine hydrochloride concentration more than ∼1.0 µM leads to a rapid decrease of the MCF (Fig. 2). The differences in MFC at each dose of these compounds for three replicates were significant (p<0.05).

To confirm the results from our primary HTS assay and to demonstrate that the decrease in PPIX fluorescence mediated by the identified compounds were not associated with their ability to alter ABCB6 expression *per se* we measured ABCB6 expression in ABCB6-overexpressing cells following treatment with each of the four compounds. As shown in Figure 2c and 2d tomatine hydrochloride and benzethonium chloride dose dependently reduced ABCB6-mediated PPIX fluorescence without affecting ABCB6 expression. Similar results were observed for verteporfin (data not shown).

Benzethonium chloride is a broad range antiseptic with a variety of antimicrobial properties [28] and a tendency to be potentially toxic [29]. The plant steroidal alkaloid α-tomatine has a variety of reported activities spanning from antifungal to immune adjuvant [30]–[32]. Verteporfin (trade name Visudyne), is a benzoporphyrin derivative used as a photosensitizer for photodynamic therapy to eliminate the abnormal blood vessels in the eye associated with conditions such as the wet form of macular degeneration [33], [34]. Piperlongumine has anti-cancer properties and selectively targets and kills cancer cells but leaving normal cells unharmed [35]. It is intriguing to note that the assay detected verteporfin, which is similar in structure to carboxylated porphyrin derivatives such as hemin and pheophorbide A, which have been shown to be ABCB6 substrates [17].

### Cytotoxicity

**Figure 3:**
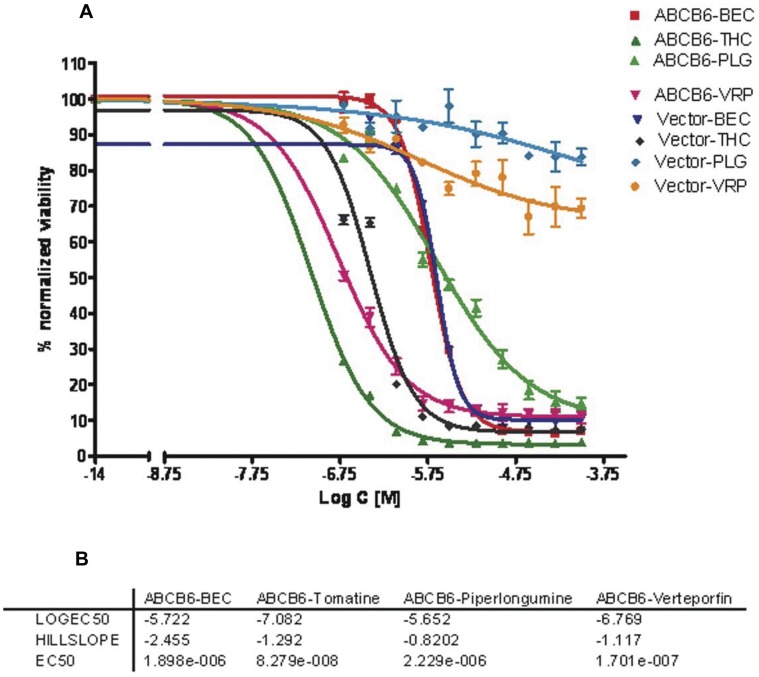
Toxicity profile for ABCB6 overexpressing and vector cells treated with the four compounds identified in the primary HTS screen. (a) Dose dependent toxicity of the four compounds in ABCB6 overexpressing and vector cells. (b) EC_50_ value for each of the compound and their corresponding hill slope values. Results represent mean +/− SD with n = 3.

A follow-up cytotoxicity assay was performed employing ABCB6 overexpressing and vector cell lines (Fig. 3). This experiment aimed to identify in principal those compounds that are not toxic at the concentrations exhibiting their functional inhibitory role by decreasing the intracellular accumulation of porphyrin mediated by ABCB6. Interestingly, the normalized viability of vector cells was reduced more than 80% when the concentration of tomatine and benzethonium chloride was >390 nM. However, viability was not reduced more than 25% even at the highest concentration (100 µM) of verteporfin and piperlongumine. The differences in percent-normalized viability at each dose of these compounds for three replicates were significant (p<0.05).

In case of ABCB6 overexpressing cells, benzethonium, verteporfin, tomatine and piperlongumine exhibited EC_50_ values of 1.9, 0.17, 0.08 and 2.23 uM, respectively. It is true that both verteporfin and tomatine are more toxic to ABCB6 cells than the other two compounds (Fig. 3). However, the selection of a less toxic compound in between benzethonium and piperlongumine is also complex. The normalized viability of ABCB6 expressing cells was not reduced significantly up to a dose of 780 nM dose of benzethonium, which was reduced by 40% at this concentration of piperlongumine.

### Topology and Homology Modeling of ABCB6

ABC transporters have a characteristic architecture that consists minimally of four domains: two transmembrane domains (TMDs), and two cytoplasmic ATP Binding domains [1]. At the sequence level, the superfamily of ABC transporters is identified by a characteristic set of highly conserved motifs that are present in the ABCs.

Although the detailed folds of the TMDs can vary, they all interact with the helical domains of the ABC domain through coupling helices that are located in the loops between TM helices [36]. In addition to the sequence comparisons of the ABC domains, the structures of the binding proteins also suggest that the hydrophobic amino acid (HAA; such as Leu, Ile, Val system) and monosaccharide (MOS; such as ribose (RBS)) branch could exhibit a distinct TMD fold. The structures of the binding proteins for ABC transporters vary in their topological threading between domains [37]–[39] and can be classified into three distinct categories (designated I, II and III, which confusingly overlaps with the nomenclature used for the TMD folds).

**Figure 4:**
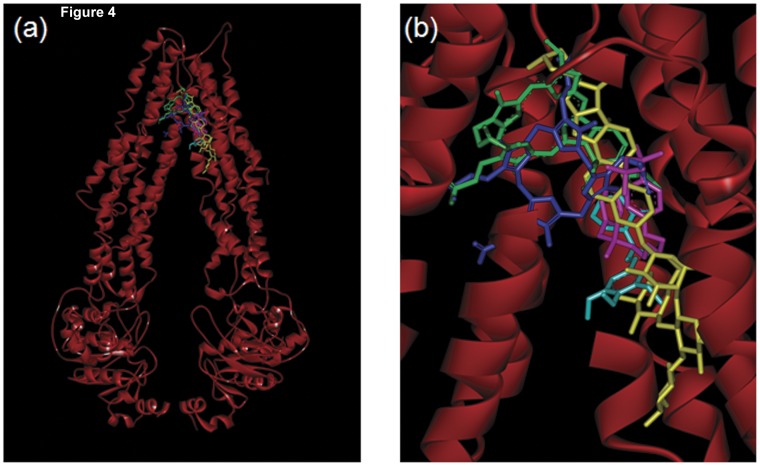
Topology and homology model of ABCB6 dimer with the docked ligands. (a) far and (b) close view of docking poses of selected ligands to the human ABCB6 transporter. Coproporphyrinogen III – blue; verteporfin – green; benzethonium chloride– magenta; piperlongumine - light blue; and tomatine hydrochloride - yellow. The Gly426-Val429, and Phe545-Pro555 parts from one ABCB6 monomer were hidden in order to better see the ligands.

The homology modeling and docking results show that the best scoring poses of the docked ligands (Fig. 4a and 4b), are positioned in the upper part of the transmembrane region, close to the corresponding binding site of the QZ59 inhibitor in the ABCB1a crystal structure [40]. This is the site where verapamil is known to bind to the ABCB1 transporter [41], [42]. While we do not intend to make quantitative predictions based on this docking model, we did notice a higher overlap between the endogenous substrate, coproporphyrinogen III, with tomatine and verteporfin, compared to the overlap with benzethonium and piperlongumine.

### Selectivity and Validation of Compounds Identified in the Primary HTS assay – Hemin Agarose Affinity Chromatography Using Mitochondrial Fractions

**Figure 5:**
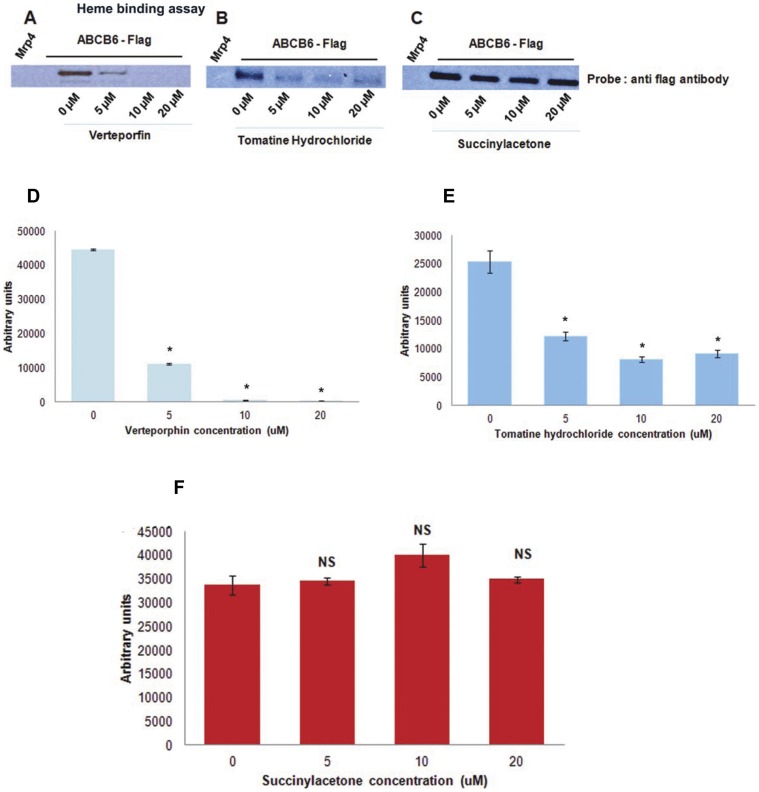
Selectivity and validation of HTS identified compounds by hemin agarose affinity chromatography. (a) verteporfin and (b) tomatine hydrochloride potently disrupt the interaction between ABCB6 and hemin-agarose compared with (c) succinylacetone. (d, e and f) image J analysis of ABCB6 band intensity treated with (d) verteporfin, (e) tomatine hydrochloride and (f) succinylacetone averaged over three independent experiments. Mitochondria isolated from K562 cells expressing ABCB6-Flag or the empty vector were incubated in the presence or absence of increasing concentration of the indicated compound and hemin-agarose and the resulting complex was immunoblotted using a monoclonal antibody to the flag-tag. Results are representative of 3 independent experiments. ‘*’ significantly different from untreated controls. P<0.05. ‘NS’ differences are non-significant compared to untreated control.

The compounds identified in the primary HTS were evaluated for their ability to act as functional inhibitors of ABCB6 using hemin-agarose affinity chromatography and mitochondrial transport assay [17]. We have previously demonstrated that these two assays when used in combination have the ability to identify substrates and potential inhibitors of ABCB6 [17]. We found that following hemin-agarose affinity chromatography, verteporfin and tomatine hydrochloride showed a dose dependent displacement of ABCB6 from hemin agarose [>50% displacement of ABCB6 at 5 µM (Fig. 5)]. In contrast, neither succinylacetone (Fig. 5) a known inhibitor of porphyrin biosynthesis nor benzethonium chloride (data not shown) one of the compounds identified in the HTS screen were capable of displacing ABCB6 even at a concentration of 20 µM. These results suggest that verteporfin and tomatine hydrochloride could be potential substrates of ABCB6 and might be capable of interacting with the same substrate-binding site as hemin.

### Selectivity and Validation of Compounds Identified in the Primary HTS Assay – Hemin Agarose Affinity Chromatography Using Purified Protein

**Figure 6:**
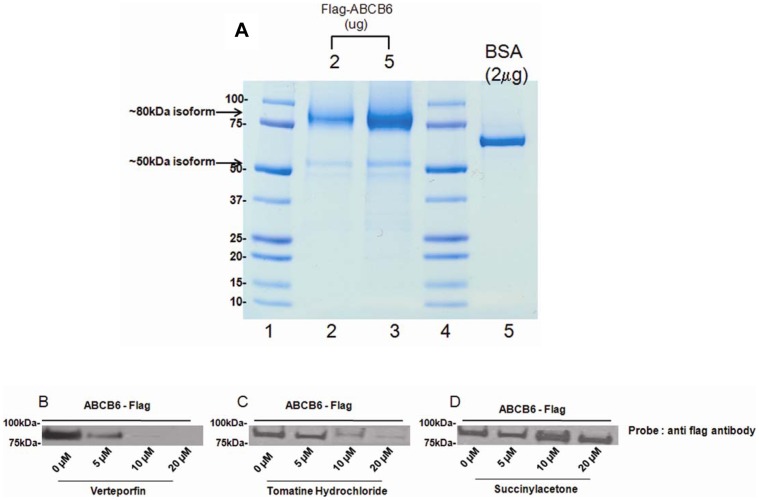
SDS-PAGE analysis of purified ABCB6 and selectivity and validation of HTS identified compounds by hemin-agarose affinity chromatography using purified ABCB6. (a) Purified ABCB6 sample was analyzed by SDS-PAGE. The figure shows coomassie brilliant blue staining of SDS gel (lane legends are 1, protein marker; 2, purified ABCB6-flag 2 µg protein; 3, purified ABCB6-flag 5 µg protein; 4, protein marker; and 5, bovine serum albumin control). b) verteporfin and (c) tomatine hydrochloride potently disrupt the interaction between purified ABCB6 protein and hemin-agarose compared with (d) succinylacetone. Three hundred nanograms of purified ABCB6-flag protein was incubated in the presence or absence of increasing concentration of the indicated compound and hemin-agarose and the resulting complex was immunoblotted using a monoclonal antibody to the flag-tag. Results are representative of three independent experiments.

To confirm hemin interaction with ABCB6 specifically and to extend the studies described above we performed hemin-agarose affinity chromatography using purified ABCB6 protein. The purification and characterization of ABCB6 is part of another manuscript that is currently in preparation and will not be discussed here in detail, except to show that the purified protein (confirmed by mass spectrometry) exists as two isforms of ∼ 50 kDa and ∼80 kDa proteins (Fig. 6a), both of which were recognized by the flag-antibody. Because the flag-antibody is fused to the c-terminal tail of ABCB6, it suggests that the two isoforms have different N-terminal sequences. We found that following hemin-agarose affinity chromatography, only the ∼80 kDa isoform was detected in immunoblots (Fig. 6b, 6c and 6d and Fig. S7 and S8), suggesting isoform specific interaction with hemin. As with the isolated mitochondria, both verteporfin and tomatine hydrochloride showed a dose dependent displacement of purified ABCB6 from hemin-agarose, while no such competition was seen with succinylacetone. Taken together results presented in Fig. 6, and Fig.S7 and S8, demonstrate for the first time that hemin directly interacts with ABCB6. Despite these results it is still quite possible that hemin interaction with ABCB6 might require additional proteins that promote hemin interaction with ABCB6. However, from the results presented here it appears that this interaction might be transient and is potentially not required once the hemin-ABCB6 complex is formed, because no interacting proteins were seen following hemin-agarose-ABCB6 affinity chromatography (Fig. S7 and S8). In addition, these results suggest that verteporfin and tomatine hydrochloride could be potential substrates of ABCB6 and might be capable of interacting with the same substrate-binding site as hemin.

The results from hemin-agarose affinity chromatography is further strengthened by the modeling studies described above which demonstrate that both tomatine and verteporfin show higher overlap with the endogenous substrate, coproporphyrinogen III (Fig. 4). Further, given that verteporfin is a carboxylated porphyrin, similar to porphyrins that have previously been demonstrated to be transported by ABCB6, these data suggest that verteporfin is a substrate for ABCB6 that competes for the same site on ABCB6 as does hemin. Thus, the identification of verteporfin in this HTS assay highlights the capability of the assay to identify not only inhibitors of ABCB6 but also potential substrates.

### Selectivity and Validation of Compounds Identified in the Primary HTS Assay – Mitochondrial Transport Assay and ATPase Activity Assay

**Figure 7:**
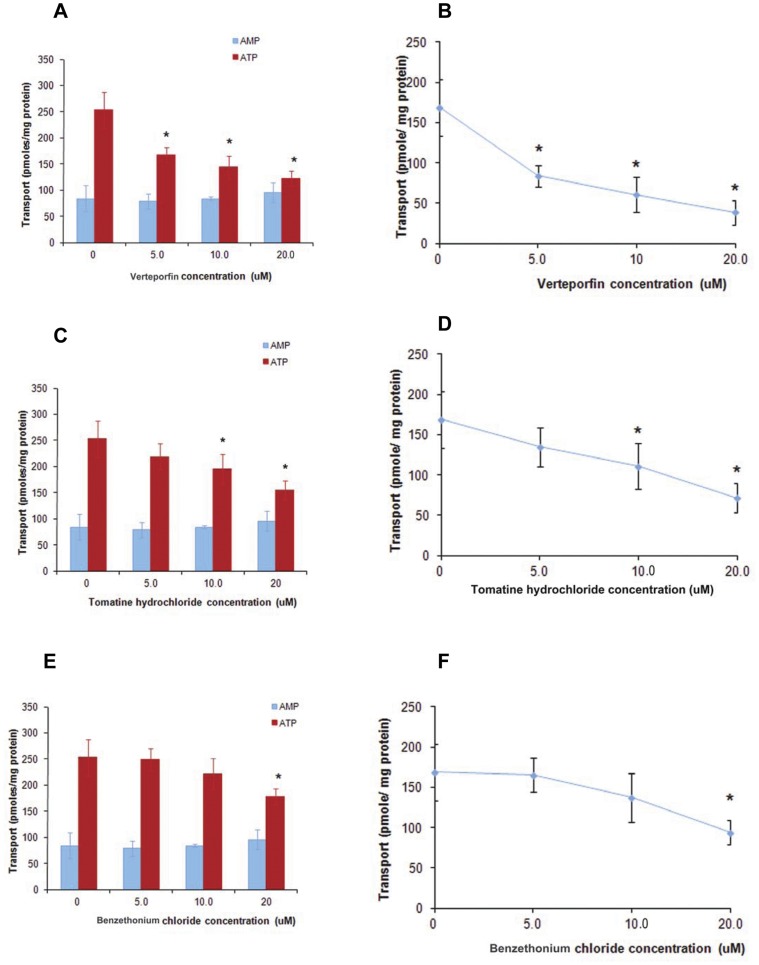
Three of the four compounds identified in the primary HTS screen compete for ABCB6 mediated hemin transport. (a) verteporfin (c) tomatine hydrochloride and (e) benzethonium chloride compete for the transport of ^14^C-labeled hemin in the presence of ATP but not in the presence of AMP in mitochondria isolated from ABCB6-overexpressing cells. (b, d, and f) Line graph showing dose dependent competition of (b) verteporfin, (d) tomatine hydrochloride and (f) benzethonium chloride for ^14^C-labeled hemin in the presence of ATP. Results representative of 3 independent experiments. ‘*’ Significantly different from untreated controls. P<0.05.

To test further the specificity of the identified compounds to inhibit ABCB6 function we evaluated the ability of verteporfin, tomatine hydrochloride and benzethonium chloride to inhibit ABCB6 mediated transport of hemin into mitochondria. We have previously demonstrated that ABCB6 is capable of transporting radioactive labeled hemin into isolated mitochondria and that this assay is capable of distinguishing ABCB6 substrates from non-substrates [17]. As shown in Figure 7, hemin uptake by isolated mitochondria was inhibited by all three compounds with verteporfin showing the highest inhibition followed by tomatine hydrochloride and benzethonium chloride. The fact that benzethonium chloride acts as a weak inhibitor of ABCB6 mediated transport of hemin into isolated mitochondria, despite the fact that it does not interfere with hemin binding to ABCB6 suggests that benzethonium chloride may function as a potential inhibitor of ABCB6 by interacting with a site that is different from that bound by hemin. These results presented above highlight the recurring theme in the transporter field where the transporters appear to have multiple substrate binding sites that promote co-operativity either in promoting or inhibiting transport of a specific substrate. In contrast to benzethonium chloride, both tomatine hydrochloride and verteporfin were capable of inhibiting transport as well as hemin interaction with ABCB6 suggesting that these compounds could be potential substrates of ABCB6.

**Figure 8:**
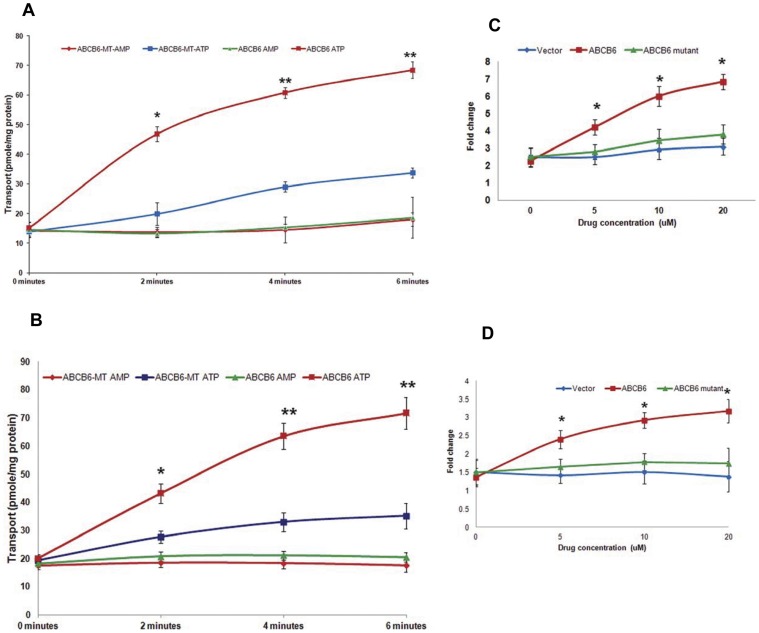
Verteporfin and tomatine hydrochloride are ABCB6 transport substrates. (a) verteporfin and (b) tomatine hydrochloride are transported by transport competent ABCB6 protein (ABCB6) in the presence of ATP which is significantly higher than transport by transport incompetent ABCB6 protein (ABCB6-MT) in the presence of ATP in mitochondria isolated from ABCB6 or ABCB6-MToverexpressing cells. Results are representative of three independent experiments. ‘*’ significantly different from ABCB6-MT and AMP treated ABCB6 expressing mitochondria; P<0.05. ‘**’ significantly different from ABCB6-MT and AMP treated ABCB6 expressing mitochondria; P<0.01. (c) and (d) vanadate sensitive ATPase activity (fold change relative to basal activity) was stimulated by (c) verteporfin and (d) tomatine hydrochloride in mitochondria from cells expressing transport competent ABCB6 protein (ABCB6) relative to mitochondria isolated from transport incompetent ABCB6 protein (ABCB6-MT). Values are means +/− SEM. ‘*’ significantly different from ABCB6-MT cells at each time point; P<0.05.

To confirm our assertion above and to demonstrate that verteporfin and tomatine hydrochloride are potential substrates of ABCB6 we performed transport assays using these compounds in functional mitochondria isolated from cells expressing either a transport competent ABCB6 protein (referred to as ABCB6 in the figure legends and Fig. 8) or a transport incompetent ABCB6 protein (referred to as ABCB6-MT) as described above in methods and [17]. As shown in Figure 8 both verteporfin (Fig. 8a) and tomatine hydrochloride (Fig. 8b) were taken up in an ATP dependent manner into functional mitochondria isolated from ABCB6 expressing cells which was significantly higher than the uptake observed using functional mitochondria isolated from ABCB6-MT cells.

We have previously demonstrated that ABCB6 mediated transport of substrates is an energy-dependent process, and that ABCB6 is a mitochondrial membrane ATPase that functions as an active drug transporter [17]. Based on these observations we hypothesized that ABCB6 might function as an ATPase during the transport of verteporfin and tomatine hydrochloride. To test this hypothesis we measured the ATPase activity of ABCB6 in the presence and absence of ATP, and the drug, using mitochondria isolated from ABCB6 expressing or ABCB6-MT expressing cells. As shown in Fig. 8 both verteporfin (Fig. 8c) and tomatine hydrochloride (Fig. 8d) stimulated vanadate-sensitive mitochondrial ATPase activity in ABCB6 expressing cells but not in ABCB6-MT expressing or vector control cells. Together results presented in Figures 7 and 8 demonstrate that verteporfin and tomatine hydrochloride could be potential substrates of ABCB6 and highlights the robustness of the HTS assay to identify not only potential inhibitors but also potential substrates of ABCB6.

There is increasing evidence that ABCB6 localizes to multiple cellular compartments including the plasma membrane [9]. Although the precise identity and the substrate specificity of ABCB6 localized to the plasma membrane are not defined, there is a potential possibility that the differentially localized ABCB6 might have overlapping substrate specificity [9]. This could lead to a possible erroneous generalization of the identified compounds as either inhibitors or substrates of ABCB6 based on the primary HTS assay. Although this is a concern that has to be considered, the secondary assays of specificity described here can eliminate these discrepancies in most cases as these secondary assays utilize isolated mitochondria and purified ABCB6 protein as opposed to whole cells used in the primary HTS assay. Further, it is interesting to note that the purified ABCB6 protein exists as two isoforms (Fig. 6a), very similar to what has been described by Paterson et al [9] as the mitochondrial and plasma membrane forms of ABCB6. In addition our results suggest that the two isoforms could have different substrate specificities as only the ∼80 kDa form of ABCB6 appears to interact with hemin but not the ∼50 kDa form. Although it is not clear at this time which of the two isoforms localize to the mitochondria, based on our earlier studies where we have shown that the ∼80 kDa form of ABCB6 interacts with heme and localizes to the mitochondria we hypothesize that the ∼80 kDa form of the ABCB6 isoform localizes to the mitochondria.

As optimized, the HTS assay is amenable to high-throughput screening applications for identification of ABCB6 modulators and in combination with the secondary assays can identify potential mitochondrial ABCB6 substrates and inhibitors. The assay is also appropriate to establish the relative potencies of active compounds. Potential pitfalls include possibility of false positives due to cytotoxic compounds or pore-forming proteins, which could allow ABCB6 function-independent leakage of PPIX, leading to a decrease in the overall cellular PPIX levels. Finally, although the assay is configured to only detect compounds that block ABCB6, the mechanism of action is not limited nor is specificity ensured. Because ABCB6 activity is dependent on ATP hydrolysis, compounds that affect ATP synthesis and accumulation could also appear as false positives. However, as with the differentially localized ABCB6, these issues can be easily resolved by secondary validation assays and specificity studies described above.

Further analysis of the compounds identified here as well as those resulting from future screening of the small Molecular Libraries Small Molecule Repository (MLPCN) is expected to yield significant inhibitors and activators of ABCB6 and potential new ABCB6 substrates. Availability of these inhibitors, activators and substrates in multiple classes will also be useful in understanding the biochemical, physiological, and clinical implications of ABCB6 in development, tumorigenesis and therapy related drug resistance.

## Supporting Information

Figure S1 **Human erythroleukemia cells (K562) expressing ABCB6 have stable expression of ABCB6.** The data show comparable ABCB6 expression in ABCB6 overexpressing cells that have undergone either 2 or 60 passages, indicating that ABCB6 expression in overexpressing cells is stable for upto 60 passages. Porin is used as the mitochondrial loading control. Figure representative of 3 independent experiments.
(TIF)

Figure S2 **ABCB6 high-throughput screening assay flow-chart.**
(TIF)

Figure S3 **The sequence of the human ABCB6 transporter (UniProt accesion code: Q9NP58) aligned with both the Val33-Thr626 and Leu684-Ala1271 parts of the sequence of the mouse ABCB1a (PDB code: 3G5U).**
(TIF)

Figure S4 **Schematic representation of ABCB6 hemin-agarose affinity chromatography.**
(TIF)

Figure S5 **Flow cytometric HTS of Prestwick Chemical Library (PCL).** The figure depicts data from a representative PCL plate demonstrating decrease in FL2 fluorescence (representing PPIX fluorescence) mediated by (a) benzethonium chloride, (b) piperlongumine, and (c) tomatine hydrochloride (tomatine). Figure (c) also demonstrates three potential activators of porphyrin biosynthesis (doxorubicin, ellipticine, and hesperidin). The Y-axis displays the amount of fluorescence (FL2 log channel representing PPIX fluorescence). The X-axis displays each well. In the assays time bins were automatically drawn around the clusters by using IDLQuery software programs, each cluster corresponds to one well. The red square represents the PPIX fluorescence in untreated ABCB6 overexpressing cells while the white square represents the PPIX fluorescence in vector control cells. The arrow highlights the decrease in PPIX fluorescence of ABCB6 expressing cells in the presence of potential inhibitors while the arrowhead highlights the increase in PPIX fluorescence in the presence of potential activators.
(TIF)

Figure S6 **Chemical structures of the identified lead compounds, benzethonium chloride (BCL), verteporfin (VRP) tomatine hydrochloride (THC) and piperlongumine (PLG).**
(TIF)

Figure S7 **SDS-PAGE analysis of purified ABCB6 and selectivity and validation of HTS identified compounds by hemin-agarose affinity chromatography using purified ABCB6.** (a) Purified ABCB6 sample was analyzed by SDS-PAAGE. The figure shows silver staining of SDS gel (lane legends are 1, protein marker; 2, purified ABCB6-flag 3 µg protein). b) verteporfin and (c) tomatine hydrochloride potently disrupt the interaction between purified ABCB6 protein and hemin-agarose. 3 µg of purified ABCB6-flag protein was incubated in the presence or absence of increasing concentration of the indicated compound and hemin-agarose and the resulting complex was analyzed on a SDS-PAGE gel. The figure shows coomassie staining of SDS gel. Results representative of two independent experiments.
(TIF)

Figure S8 **Selectivity and validation of HTS identified compounds by hemin-agarose affinity chromatography using purified ABCB6.** a) verteporfin and (b) tomatine hydrochloride potently disrupt the interaction between purified ABCB6 protein and hemin-agarose. Three hundred nanograms of purified ABCB6-flag protein was incubated in the presence or absence of increasing concentration of the indicated compound and hemin-agarose and the resulting complex was immunoblotted using a monoclonal antibody to the flag-tag. Results show the entire immunoblot. Results are representative of three independent experiments.
(TIF)

Figure S9 **Viability of K562 cells exposed to DMSO.** DMSO at a concentration of 1% does not affect K562 cell survival following 24 hr exposure.
(TIF)
