# Supplementary figures and images for: A Novel Flow Cytometric HTS Assay Reveals Functional Modulators of ATP Binding Cassette Transporter ABCB6
Source: PLoS One. 2012 Jul 10;7(7):e40005. doi: 10.1371/journal.pone.0040005 (PMC3393737; doi:10.1371/journal.pone.0040005)

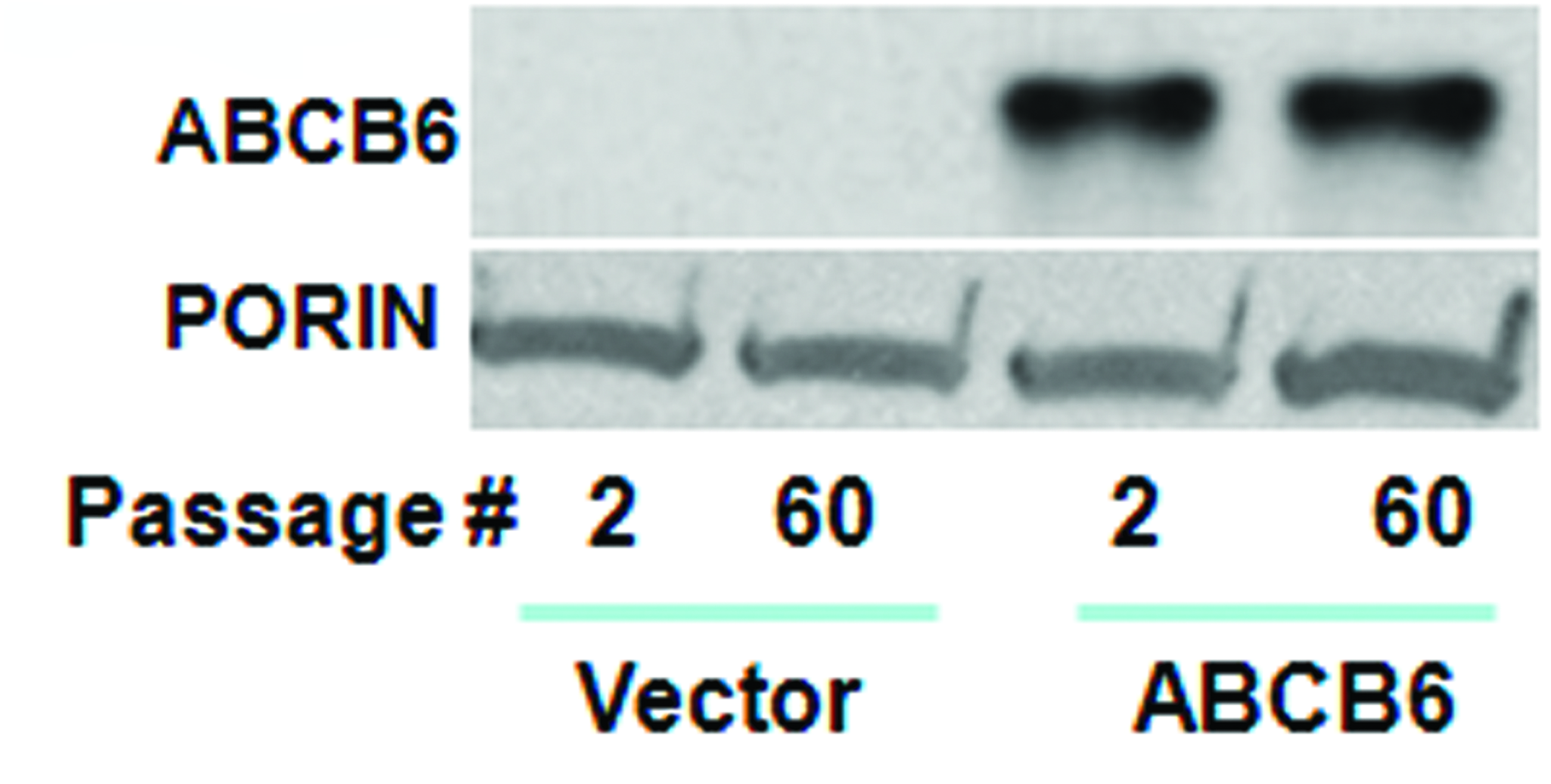

Supplement: Figure S1 — Human erythroleukemia cells (K562) expressing ABCB6 have stable expression of ABCB6. The data show comparable ABCB6 expression in ABCB6 overexpressing cells that have undergone either 2 or 60 passages, indicating that ABCB6 expression in overexpressing cells is stable for upto 60 passages. Porin is used as the mitochondrial loading control. Figure representative of 3 independent experiments. (TIF) [file pone.0040005.s001.tif]

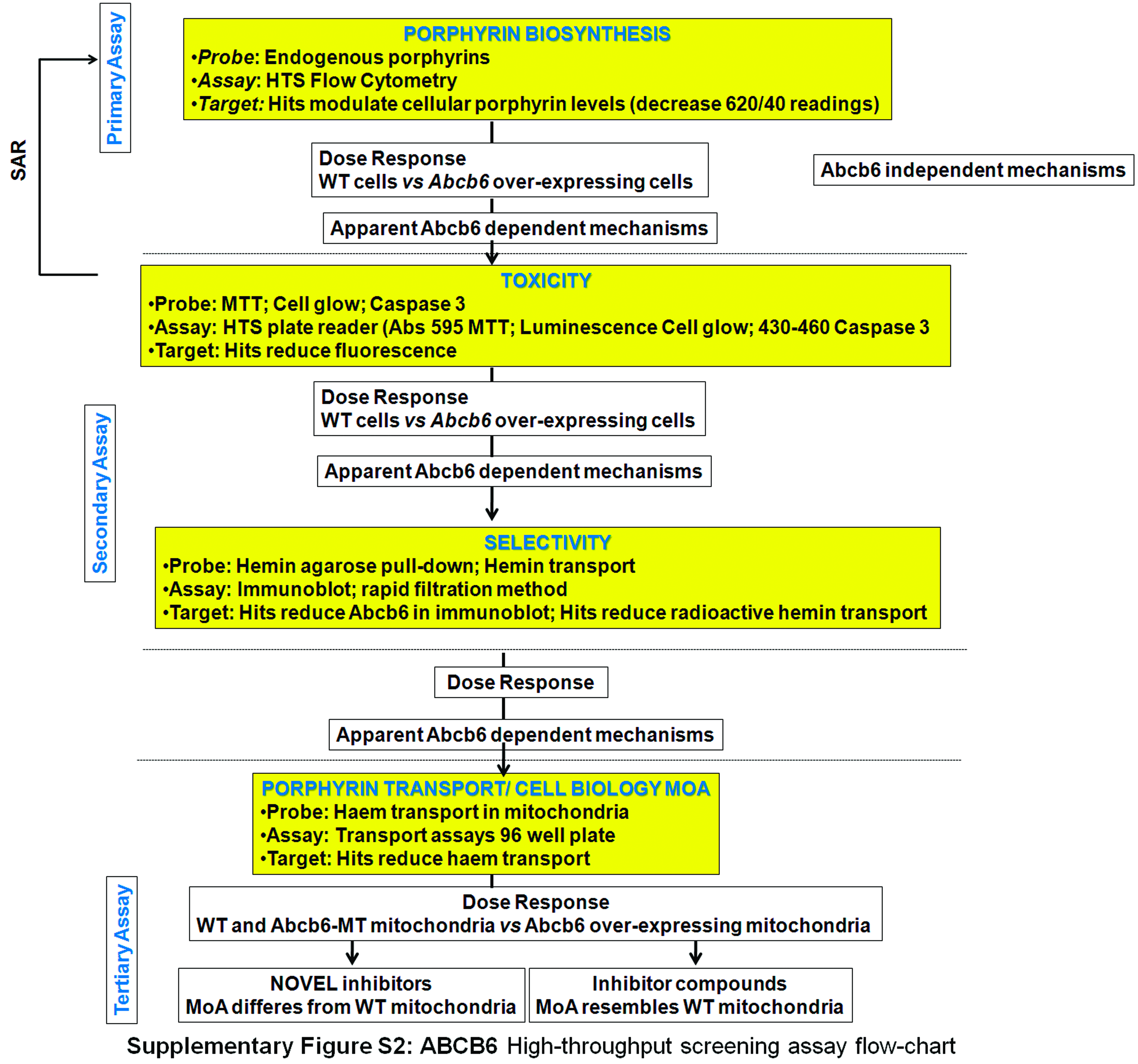

Supplement: Figure S2 — ABCB6 high-throughput screening assay flow-chart. (TIF) [file pone.0040005.s002.tif]

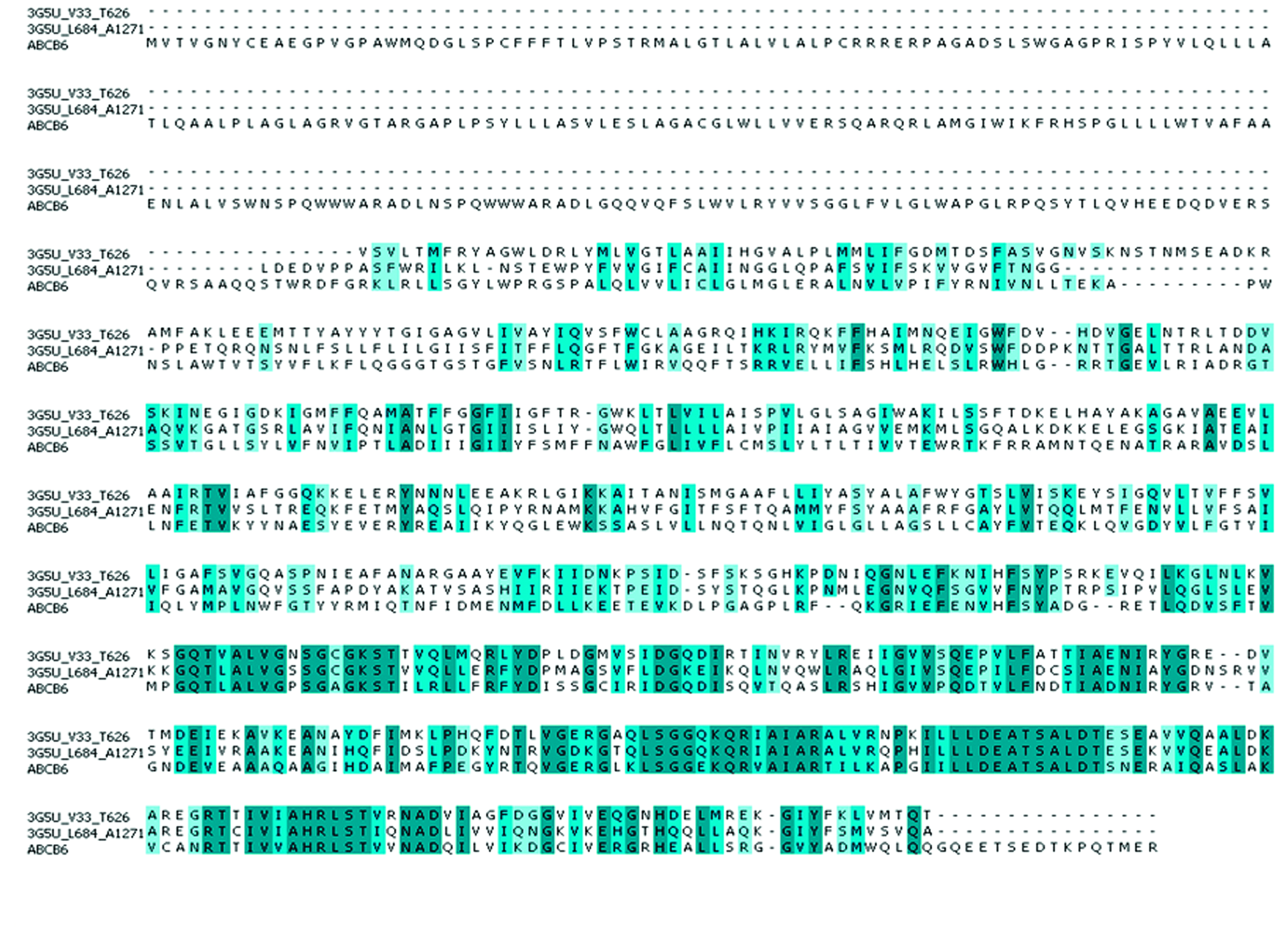

Supplement: Figure S3 — The sequence of the human ABCB6 transporter (UniProt accesion code: Q9NP58) aligned with both the Val33-Thr626 and Leu684-Ala1271 parts of the sequence of the mouse ABCB1a (PDB code: 3G5U). (TIF) [file pone.0040005.s003.tif]

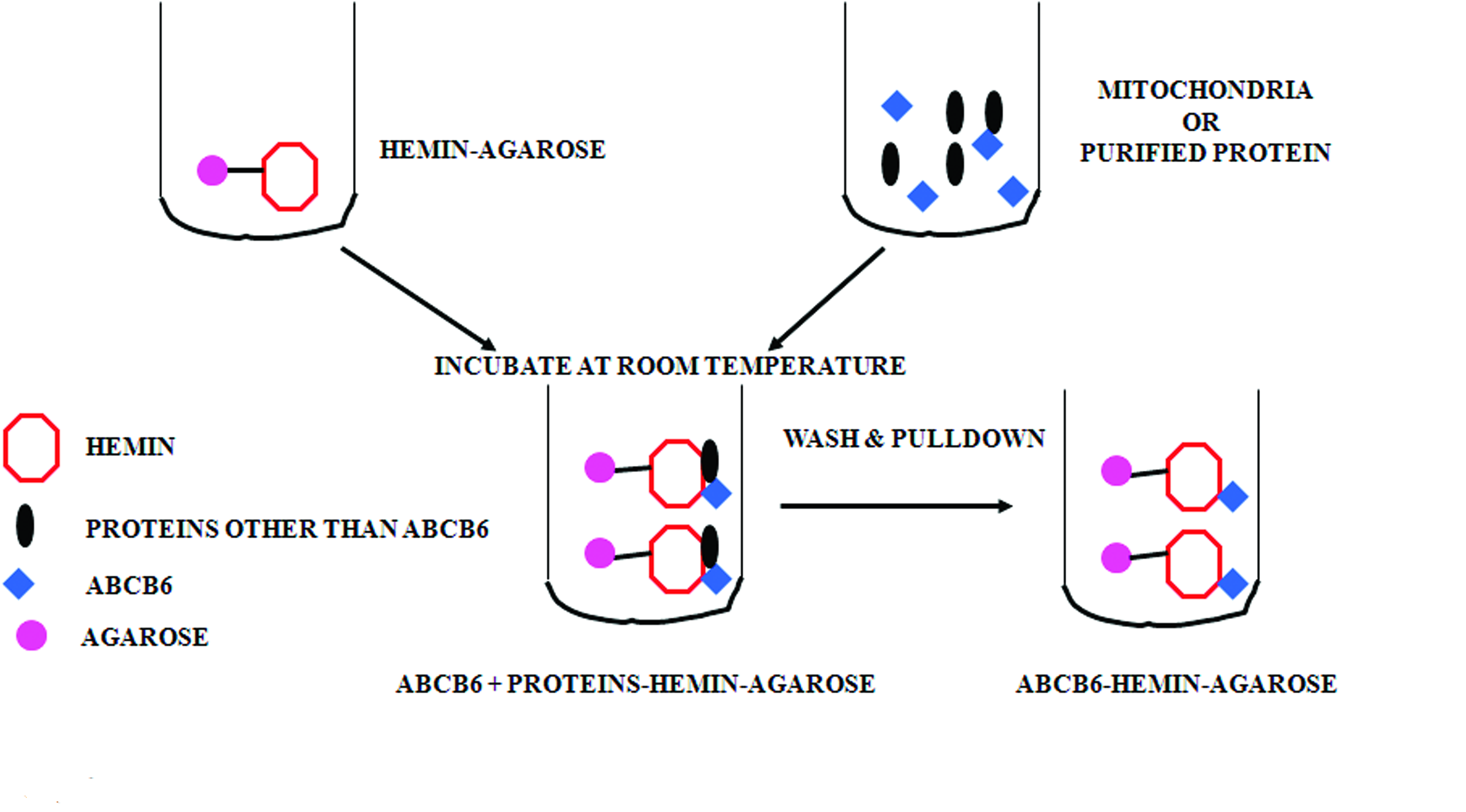

Supplement: Figure S4 — Schematic representation of ABCB6 hemin-agarose affinity chromatography. (TIF) [file pone.0040005.s004.tif]

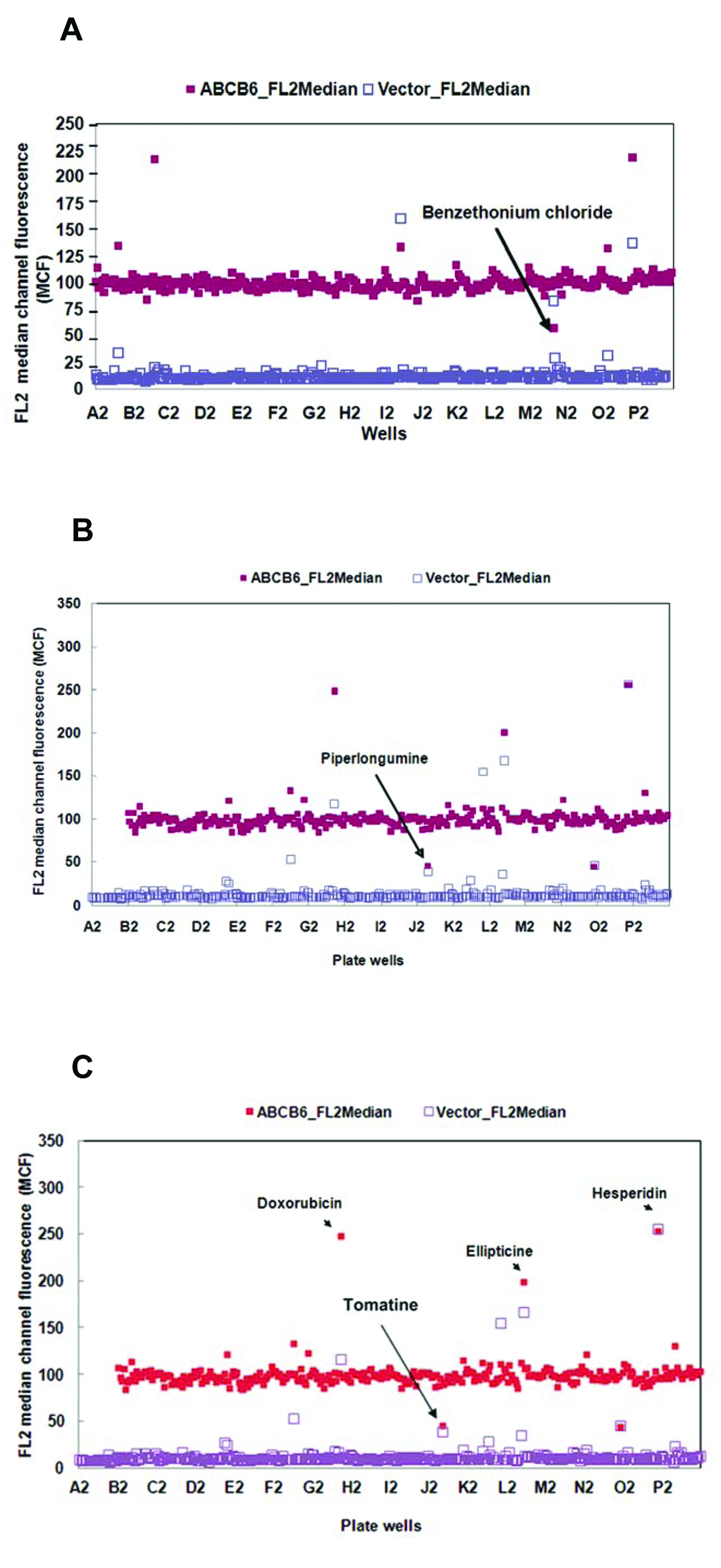

Supplement: Figure S5 — Flow cytometric HTS of Prestwick Chemical Library (PCL). The figure depicts data from a representative PCL plate demonstrating decrease in FL2 fluorescence (representing PPIX fluorescence) mediated by (a) benzethonium chloride, (b) piperlongumine, and (c) tomatine hydrochloride (tomatine). Figure (c) also demonstrates three potential activators of porphyrin biosynthesis (doxorubicin, ellipticine, and hesperidin). The Y-axis displays the amount of fluorescence (FL2 log channel representing PPIX fluorescence). The X-axis displays each well. In the assays time bins were automatically drawn around the clusters by using IDLQuery software programs, each cluster corresponds to one well. The red square represents the PPIX fluorescence in untreated ABCB6 overexpressing cells while the white square represents the PPIX fluorescence in vector control cells. The arrow highlights the decrease in PPIX fluorescence of ABCB6 expressing cells in the presence of potential inhibitors while the arrowhead highlights the increase in PPIX fluorescence in the presence of potential activators. (TIF) [file pone.0040005.s005.tif]

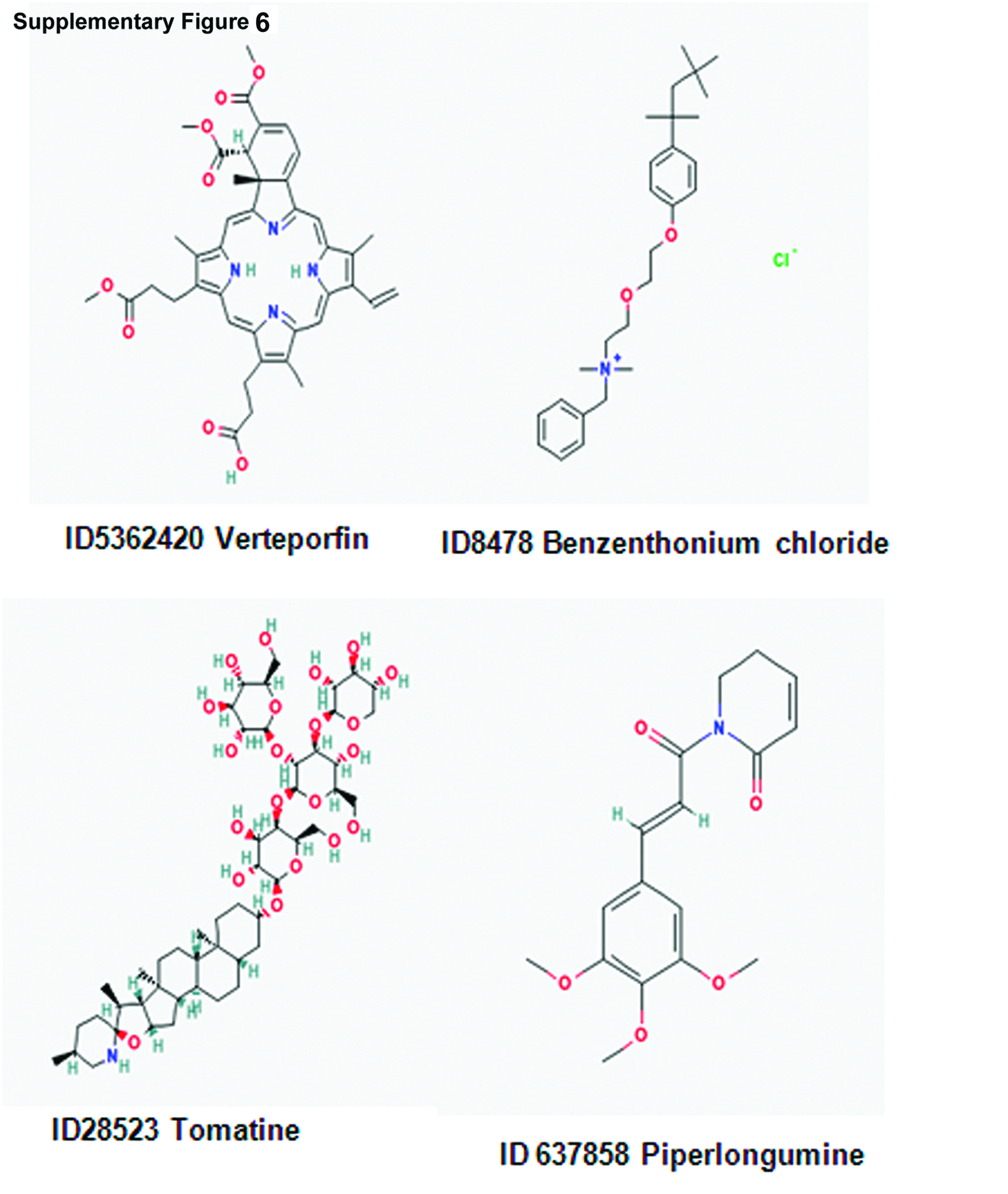

Supplement: Figure S6 — Chemical structures of the identified lead compounds, benzethonium chloride (BCL), verteporfin (VRP) tomatine hydrochloride (THC) and piperlongumine (PLG). (TIF) [file pone.0040005.s006.tif]

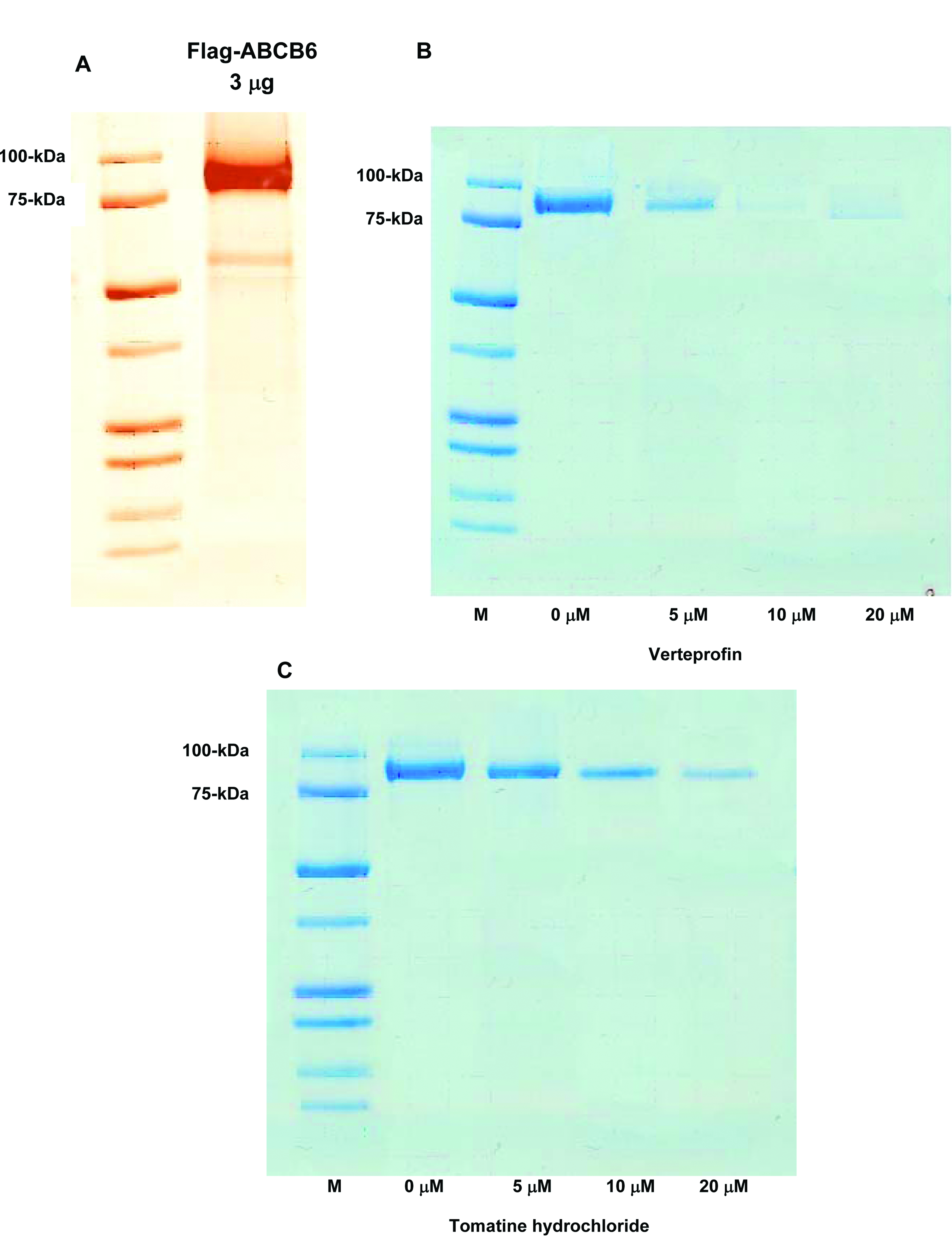

Supplement: Figure S7 — SDS-PAGE analysis of purified ABCB6 and selectivity and validation of HTS identified compounds by hemin-agarose affinity chromatography using purified ABCB6. (a) Purified ABCB6 sample was analyzed by SDS-PAAGE. The figure shows silver staining of SDS gel (lane legends are 1, protein marker; 2, purified ABCB6-flag 3 µg protein). b) verteporfin and (c) tomatine hydrochloride potently disrupt the interaction between purified ABCB6 protein and hemin-agarose. 3 µg of purified ABCB6-flag protein was incubated in the presence or absence of increasing concentration of the indicated compound and hemin-agarose and the resulting complex was analyzed on a SDS-PAGE gel. The figure shows coomassie staining of SDS gel. Results representative of two independent experiments. (TIF) [file pone.0040005.s007.tif]

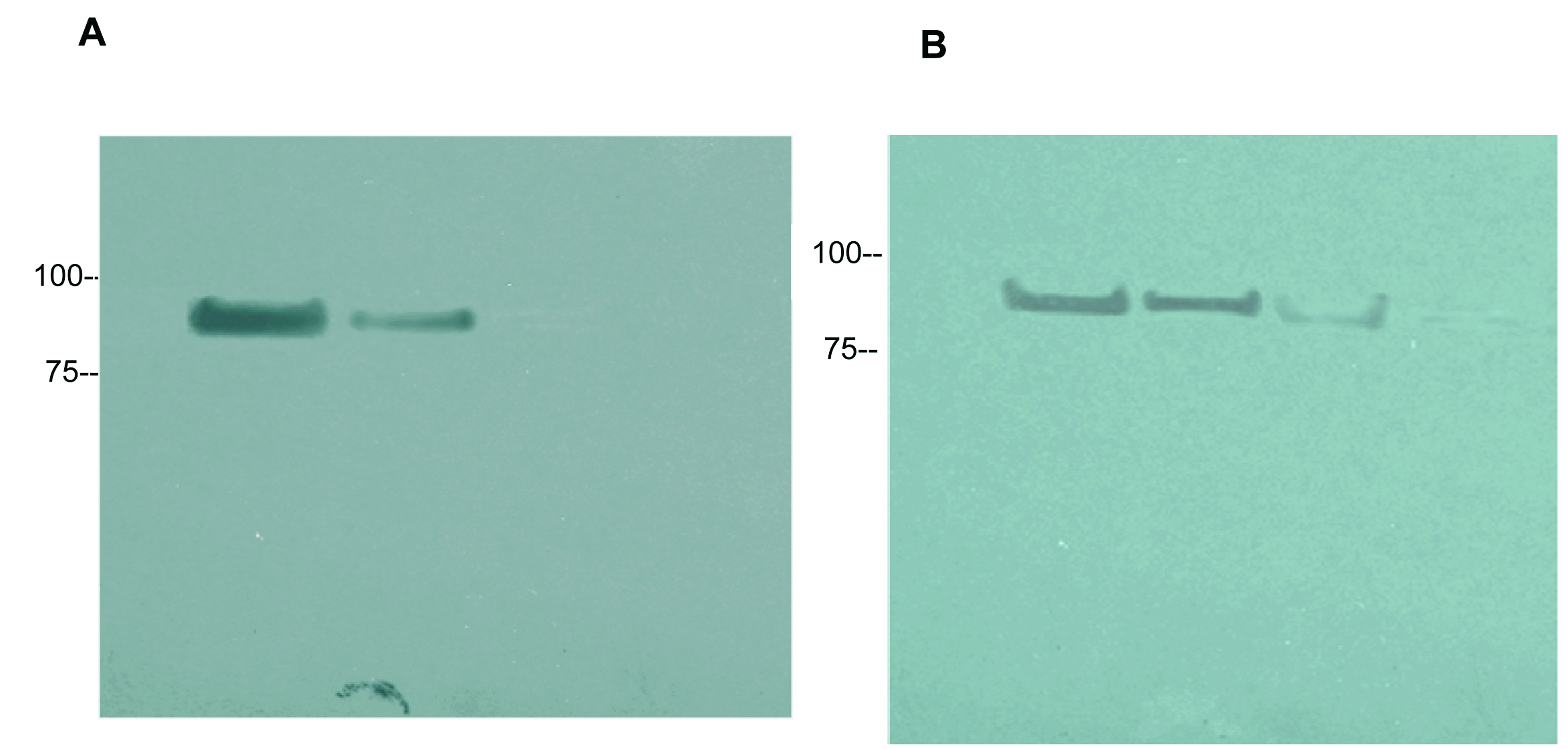

Supplement: Figure S8 — Selectivity and validation of HTS identified compounds by hemin-agarose affinity chromatography using purified ABCB6. a) verteporfin and (b) tomatine hydrochloride potently disrupt the interaction between purified ABCB6 protein and hemin-agarose. Three hundred nanograms of purified ABCB6-flag protein was incubated in the presence or absence of increasing concentration of the indicated compound and hemin-agarose and the resulting complex was immunoblotted using a monoclonal antibody to the flag-tag. Results show the entire immunoblot. Results are representative of three independent experiments. (TIF) [file pone.0040005.s008.tif]

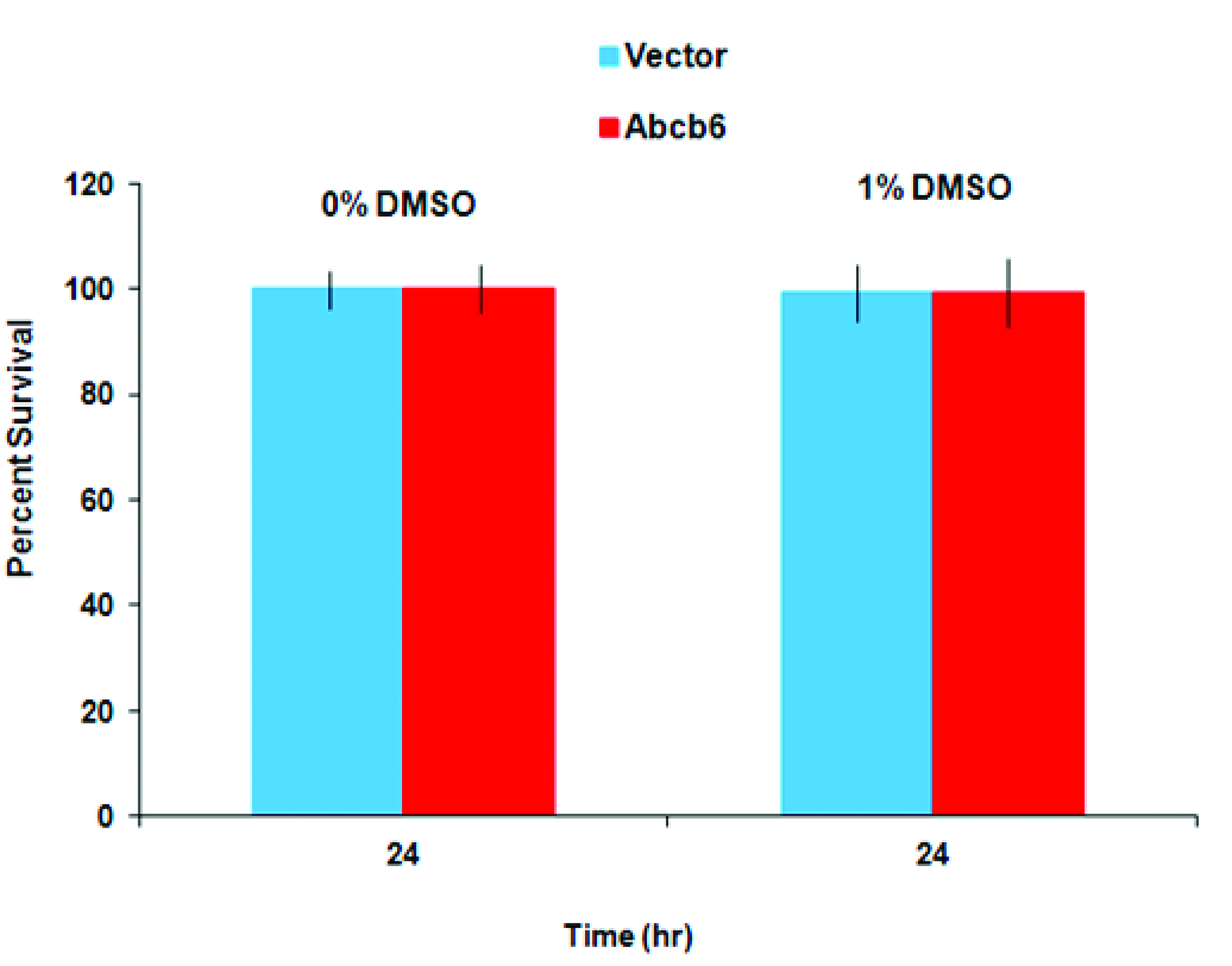

Supplement: Figure S9 — Viability of K562 cells exposed to DMSO. DMSO at a concentration of 1% does not affect K562 cell survival following 24 hr exposure. (TIF) [file pone.0040005.s009.tif]
